# Cyberknife Radiosurgery for Trigeminal Neuralgia

**DOI:** 10.7759/cureus.6014

**Published:** 2019-10-28

**Authors:** Pantaleo Romanelli, Alfredo Conti, Irene Redaelli, Anna Stefania Martinotti, Achille Bergantin, Livia Corinna Bianchi, Giancarlo Beltramo

**Affiliations:** 1 Neurosurgery, Cyberknife Center, Centro Diagnostico Italiano, Milano, ITA; 2 Neurosurgery, University of Messina, Messina, ITA; 3 Medical Physics, Cyberknife Center, Centro Diagnostico Italiano, Milano, ITA; 4 Radiation Oncology, Cyberknife Center, Centro Diagnostico Italiano, Milano, ITA

**Keywords:** trigeminal neuralgia, pain, stereotactic radiosurgery, robotic, image-guided, cyberknife

## Abstract

Introduction

Image-guided robotic radiosurgery is an emerging minimally-invasive treatment option for trigeminal neuralgia (TN). Our group has treated 560 cases up to date, and report here the clinical outcomes of 387 treatments with three years follow-up. This study represents the largest single-center experience on CyberKnife radiosurgery for the treatment of TN so far reported.

Methods

CyberKnife radiosurgery treatment was offered to patients with drug-resistant TN, after the failure of other treatments or refusal of invasive procedures. A second treatment was offered to patients with a poor response after the first treatment or with recurrent pain. Treatment protocol required the non-isocentric delivery of 60 Gy prescribed to the 80% isodose to a 6 mm retrogasserian segment of the affected trigeminal nerve. Retreatments typically received 45 Gy, again prescribed to the 80% isodose. The final plan was developed accordingly to individual anatomy and dose distribution over the trigeminal nerve, gasserian ganglion, and brainstem. Clinical outcomes such as pain control and hypoesthesia/numbness have been evaluated after 6, 12, 24, and 36 months.

Results

Our group has treated 527 patients with Cyberknife radiosurgery at Centro Diagnostico Italiano (CDI), Milan, Italy, during the last decade. A minimum follow-up of six months was available on 496 patients. These patients received 560 treatments: 435 patients (87.7%) had a single treatment, 60 patients (12.1%) had two treatments, and one patient (0.2%) had five treatments (two on the right side, three on the left side). Twenty four patients had multiple sclerosis (4.8%). Four hundred and forty-three patients (84%) received the treatment without previous procedures, while 84 patients (16%) underwent radiosurgery after the failure of other treatments. A neurovascular conflict was identified in 59% of the patients. Three hundred and forty-three patients (receiving a total of 387 treatments) had a minimum of 36 months follow up. Pain relief rate at 6, 12, 18, 24, 30 and 36 months was respectively 92, 87, 87, 82, 78 and 76%. Forty-four patients out of 343 (12.8%) required a second treatment during the observed period. At 36 months post-treatment, 21 patients (6,1%) reported the presence of bothering facial hypoesthesia. Eighteen patients out of 21 (85.7%) developed this complication after a repeated treatment.

Conclusions

Frameless image-guided robotic radiosurgery in experienced hands is a safe and effective procedure for the treatment of TN, providing excellent pain control rates in the absence of major neurological complications. Repeated treatments due to recurrent pain are associated with restored pain control but at the price of a higher rate of sensory complications.

## Introduction

Trigeminal neuralgia (TN) is the most common craniofacial pain syndrome. Irradiation of the cisternal portion of the nerve by stereotactic radiosurgery (SRS) is an effective alternative therapeutic option to interrupt the trigeminal nociceptive pathways [1-12]. SRS is therefore offered to medically refractory TN patients, to patients with a high medication burden, and to those who have failed or refused surgery or are not suitable for major surgery [1,2,6,13]. Gamma Knife (GKS) frame-based radiosurgery using a single isocenter provides the bulk of the literature currently available about radiosurgical treatment of TN [1-12]. Typically GKS provides up to 92% pain relief within the first year, but a significant rate of pain relapse within five years [1-6]. Frameless radiosurgical treatment of TN using the CyberKnife (Accuray Inc., Sunnyvale, California), an image-guided robotic system enabling homogeneous irradiation of an extended segment of the trigeminal nerve using non-isocentric irradiation delivery was first reported by John Adler's group at Stanford in 2003 [14]. 

About a decade ago, the senior author of this paper (PR) collecting the early years' experience, developed a TN CyberKnife radiosurgery protocol which has been consistently used at Centro Diagnostico Italiano (CDI), Milan, Italy, treating 560 TN cases up to date. We have recently published a prospective study reporting the clinical outcomes after CyberKnife radiosurgery for TN on a cohort of 138 patients with follow-up extending over five years [13]. This was the first single-center paper studying the long-term effects of CyberKnife radiosurgery on pain control and sensory outcomes on a homogenous cohort of patients selected and treated according to a single protocol. Previous reports about CyberKnife radiosurgery for TN were all retrospective studies limited either by small numbers and inhomogeneous imaging/ treatment features or, in case of multicentric studies, by the lack of common protocols regarding the selection of patients, the planning methods (non-isocentric versus isocentric), the target volumes or the doses delivered [14-23].

The CDI prospective trial has shown favorable results of CyberKnife radiosurgery for TN on 138 patients with follow-up extending to five years [13]. Clinical outcomes reported by this study indicate that pain control and sensory complication appear to stabilize after three years. Therefore, we retrospectively analyzed the clinical results in a much larger cohort of patients, focusing on the development of pain control and sensory complications in 343 patients during the first 36 months following the treatment. 

## Materials and methods

Setting and study design

Data were collected and retrospectively analyzed at CyberKnife Center, Centro Diagnostico Italiano( CDI), Milan. 

Participants

The study includes patients fulfilling TN criteria, according to the International Headache Society, who also received image-guided robotic radiosurgical treatment for medically resistant pain between 2009 and 2018. 

Variables

Pain was classified as typical (TN1), if described as sharp, shooting, electrical shock-like, with pain-free intervals between the attacks. It was classified as atypical (TN2) in patients describing the pain as an aching, throbbing, or burning pain, for more than 50% of the time and constant in nature (constant background pain being the most significant attribute). 

Radiosurgical treatment 

Patients were treated with SRS using a CyberKnife G3 (Accuray Inc., Sunnyvale, CA) until October 2011 and a Cyberknife VSI (Accuray Inc., Sunnyvale, CA) from October 2011 onwards. Before treatment, a thermoplastic mask was individually attached to each patient for treatment immobilization before high-resolution thin-slice (0.6 mm) computed tomography (CT) with contrast medium. The treatment planning was carried out on the CT with co-registered constructive interference in steady-state (CISS) and T1 weighted magnetization-prepared rapid gradient echo (MPRAGE) MRI images using MultiPlan (Accuray Inc., Sunnyvale, CA, current version 5.2.1). 

The treatment planning, including target volume delineation, dose prescription, and optimization of the dose distribution, was executed by a team of two physicians (a radiation oncologist and a neurosurgeon) and a medical physicist. A second independent check of the plan was accomplished by a medical physicist after the physician's approval. An inverse treatment planning algorithm was used to generate steep dose gradients by means of non-isocentric beam delivery with up to 1600 incident beam positions, thereby allowing optimal tumor coverage and minimal dosage to organs and tissues at risk for late radiation damage.

Target and dose selection 

Treating physicians co-registered the CT and MRI datasets and checked the quality of co-registration visually using multiple views and transparency tools of the treatment planning system (TPS) (Multiplan, Accuray Inc.) in the three projections. After that, they identified the gasserian ganglion and the retrogasserian portion of the trigeminal nerve on the MRI and a bony canal on the edge of the petrous bone clearly identifying the ostium of the Meckel’s cave. This point can be identified on CT in axial view and then checked on the sagittal and coronal view using a crosshair. An elongated 6 mm retrogasserian target was drawn on three slices along the major axis of the trigeminal nerve. The brainstem, the gasserian ganglion, the 7th and 8th cranial nerves, the cochlea, the labyrinth, and the vessels close to the trigeminal nerve were specifically delineated as critical structures to minimize radiation dose with the inverse planning algorithm. Other critical volumes, including the eyes, the lenses, and eventually the dental prosthesis, were also delineated to prevent incident beams passing through them. Furthermore, two or three tuning structures were delineated to restrict isodose distribution outside the target within a precise distance. The "Full Path Set" for intracranial targets, a specifically defined path of the robot with a typical source-axis distance (SAD) of 800 mm, and a fixed collimator of 5 mm diameter, defined at SAD 800 mm, were selected for the treatment plan. Dose calculation was performed using the ray tracing algorithm. The prescription dose was 60-65 Gy in single fraction for the first treatment and 40-45 Gy in single fraction for retreatments, prescribed to the 80-90% isodose line. Once the calculation was performed, we verified that a 6 mm segment of the trigeminal nerve was included in the prescription isodose line. The maximal length and the volume of the nerve were determined by individual anatomy (length of the nerve and the relative dose received by the brainstem) and eventually included in the prescription isodose line. Dose constraints for the brainstem were as follows: a volume equal or less than 1 cm³ could receive a dose of 10 Gy, with a maximum point dose (0.035 cm³) of 30% of the prescription dose. A mean dose <20 Gy and a maximum point dose <35 Gy were accepted for the gasserian ganglion. Maximum doses for cranial nerves and acoustic apparatus were 6 Gy and 4 Gy, respectively. For the vessels, a specific dose constraint was not defined. The treatment plan was developed during the same day of imaging acquisition. Overall the planning procedure required 45-90 minutes. Treatment was delivered the day after imaging and planning.​​​​​​​

Patient setup and treatment delivery 

Patients were set up on the treatment couch utilizing a thermoplastic mask that had been custom-formed at the time of simulation, and the 6D skull tracking mode was used for treatment. The target locating system (TLS) compares orthogonal kV X-ray pairs, so-called live images, obtained during the patient set up, to the planning system-generated digitally reconstructed radiographs (DRRs), obtained from the CT scan. At the start of the treatment, the patient was aligned using an adjustable five-degrees-of-freedom (5DOF) treatment table. The purpose of this initial alignment was to reduce the corrections that will be required from the robotic manipulator below maximum limits, which are +/- 10 mm in each direction and +/- 1° about each axis. For each live image acquisition, the TLS determined the additional translational and rotational corrections, which were compensated to redirect the beams on the target. For the trigeminal neuralgia treatment, the frequency of live images acquisition was 30-45 sec for all patients, to reduce intrafraction inaccuracy. The treatment delivery, on average, took about 50 minutes.

Quality assurance

The targeting accuracy of the 6D skull tracking mode was verified performing an end-to-end (E2E) test, using a dedicated Accuray anthropomorphic phantom with a specific insert (Ballcube II) for EBT3 ​Gafchromic films. The phantom was subjected to the entire treatment process adopted for patients: ​​​​​​CT scan with immobilization device and standard acquisition parameters, target volume delineation, plan generation, treatment delivery. A special treatment plan ​​​​​​was constructed with the full path Set, 30 fixed collimator cone, in an isocentric beams geometry. The target dose was prescribed at 70% isodose line. Such test was performed by medical physicists on a monthly basis; film analysis was performed by Accuray proprietary software. The mean total targeting error (TTE) was measured as 0.41 +/- 0.11 mm; the median was 0.39 mm. Assessments of MR image quality were performed on a semiannual basis using dedicated phantoms and included: geometric accuracy, slice thickness and intervals accuracy, slice position accuracy, image intensity uniformity, uniformity of signal-to-noise ratio, percent signal ghosting, low-contrast object detectability, and geometric distortion. The accuracy of the registration algorithm was verified, calculating the target registration error (TRE) as the average residual error between the same marker points identified on the different imaging modalities. TRE needs to be less than or equal to 1 mm.

Data sources

Clinical and treatment data were collected in a digital archive. Follow-up information was retrospectively obtained by outpatient clinical evaluation at defined time intervals.

Bias

To avoid inconsistent interpretation, clinical results were evaluated according to numerical scales, and a score was assigned at each patient at different time points. 

Assessment of outcome

Clinical follow up was carried out regularly on all the patients performing detailed assessments of pain intensity and new onset of hypoesthesia and/or paresthesias. The end-points analyzed were: 1) effects on pain scores, 2) effects on medication, 3) occurrence of sensory disturbance, 4) onset and intensity of recurrent pain. 

Quantitative variables

Pain level was scored using the Barrow Neurological Institute scale (BNI; class I: no trigeminal pain, no medication; II: occasional pain, not requiring medication; IIIa: no pain, continued medication; IIIb: controlled with medication; IV: some pain, not adequately controlled with medication; V: severe pain, no pain relief). For hypoesthesia evaluation, we used the BNI facial hypoesthesia scale (class I: no facial numbness; II: mild facial numbness, not bothersome; III: facial numbness, somewhat bothersome; IV: facial numbness, very bothersome). We considered the pain response as sufficient or inadequate after SRS if the patients reported BNI pain scores I-IIIb and IV-V, respectively. Similarly, we defined a clinically not significant or bothersome numbness as BNI grades I-II and III-IV, respectively.

Statistical methods

The overall pain-free interval was investigated using Kaplan Meier analysis for 6, 12, 24, and 36 months outcomes. The treatment parameters were reported as median and mean ± standard deviation (SD). IBM SPSS (Version 25.0. Armonk, NY: IBM Corp.) was used for statistical analysis. A p-value < 0.05 was considered as significant.

## Results

Participants

Five hundred and twenty-seven patients with TN underwent CyberKnife radiosurgery during the last 12 years. A minimum follow-up of six months was available for 496 patients. These patients received 560 treatments: 435 patients (87,7%) received a single treatment, 60 patients (12.1%) received two treatments, one patient (0.2%) received five treatments (two on the right side, three on the left side). Three hundred and forty-three patients, receiving a total of 387 treatments, had a minimum of 36 months follow up. Their clinical outcomes have been retrospectively reviewed.

Descriptive data

Pre-operatively, all patients had severe pain with a numerical rating scale (NRS) score of >7 (median score 9) and were in BNI class IV (33%) or V (67%). The pain was classified as TN1 in 91%. The pain was on the right side in 55.6% and on the left side in 44.4% of the patients. Trigeminal branches involved included V3 involvement in 45%, V2 involvement in 23%, V1 involvement in 7%, V2-V3 21%, V1-V2-V3 4%. All patients had taken medications for an average of 4.3 years (range, 11 months-17 years) before treatment. Overall, 24 patients (4.8%) had multiple sclerosis. CyberKnife radiosurgery was the first procedure in 412 of 527 patients (83.1%) while the remaining 84 patients (16.9%) underwent radiosurgery after the failure of other procedures including microvascular decompression (MVD) (27 patients, 5.4%) or percutaneous treatments such as glycerol injection, thermal ablation or balloon compression (57 patients, 11.5%). A neurovascular conflict was identified in 59% of the patients. Pain control and hypoesthesia rates became substantially stable three years after the first treatment in our previously published prospective report [11]. To further assess this finding, we analyzed the clinical outcomes during the first three years after image-guided robotic radiosurgery for TN, which included 343 patients receiving a total of 387 treatments. The mean age of this group of patients was 63.7±15.8 years.

Target and treatment data

All treatments were performed in a single fraction using a non-isocentric technique. A median dose of 60 ±4.8 Gy was prescribed to the 82±3% median isodose (mean dose and mean prescribed isodose being, respectively, 57.7 Gy and 82.6%), with a median and mean maximum dose (Dmax) of, respectively, 71.3 Gy and 69.8 Gy. The median target volume was 27 ±16.5 mm³, with a mean target volume of 30 mm³. In 49% of patients (169/343 pts.), because of a short cisternal segment of the trigeminal nerve, the final target turned out to be shorter (4 to 5 mm) with a median volume of 25 mm^3^. The median new conformation index (nCI) was 1.86 (mean 2.1 ± 2.3); the median homogeneity index (HI) was 1.25. The median number of beams was 105 (range, 90-110); median number of nodes was 87 (range, 85-90). Treatment time ranged from 45 to 55 minutes, with beam-on time ranging from 15 to 21 minutes.

Pain control 

Based on previous findings, pain control and hypoesthesia rates became substantially stable three years after the first treatment [13]. Therefore, we focused on the pain and hypoesthesia outcomes of 343 patients (receiving a total of 387 treatments) during the 36 months following the first CyberKnife procedure. Pain relief rate at 6, 12, 18, 24, 30 and 36 months was respectively 92, 87, 87, 82, 78 and 76%. 

**Figure 1:**
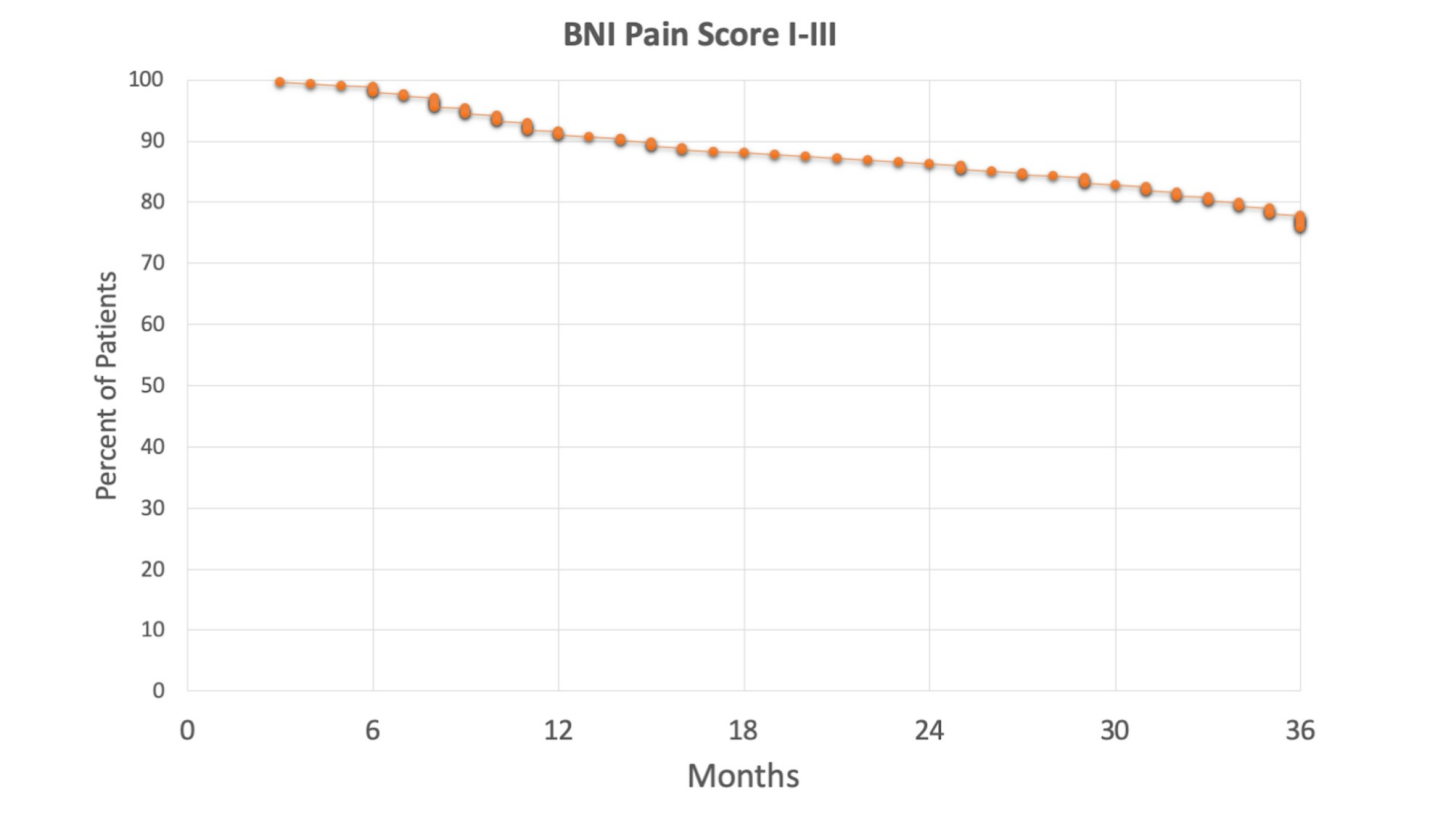
Actuarial pain control rates (Barrow Neurological Institute [BNI] Pain Score I-III) of patients after the first treatment

After a median time of three weeks, significant pain relief (a decrease in VAS score of >5) was achieved in 67% of the patients. The maximum rate of pain relief was achieved after six months (92%), with a slow decrease over time. At this time, the rate of patients free of pain without medications (BNI pain class I) was 61 %, rising to 72% after one year. Overall 78% were completely pain-free (BNI classes I and II) after one year. Figure 1 shows the actuarial rate of pain control over the first three years after the treatment.

**Figure 2:**
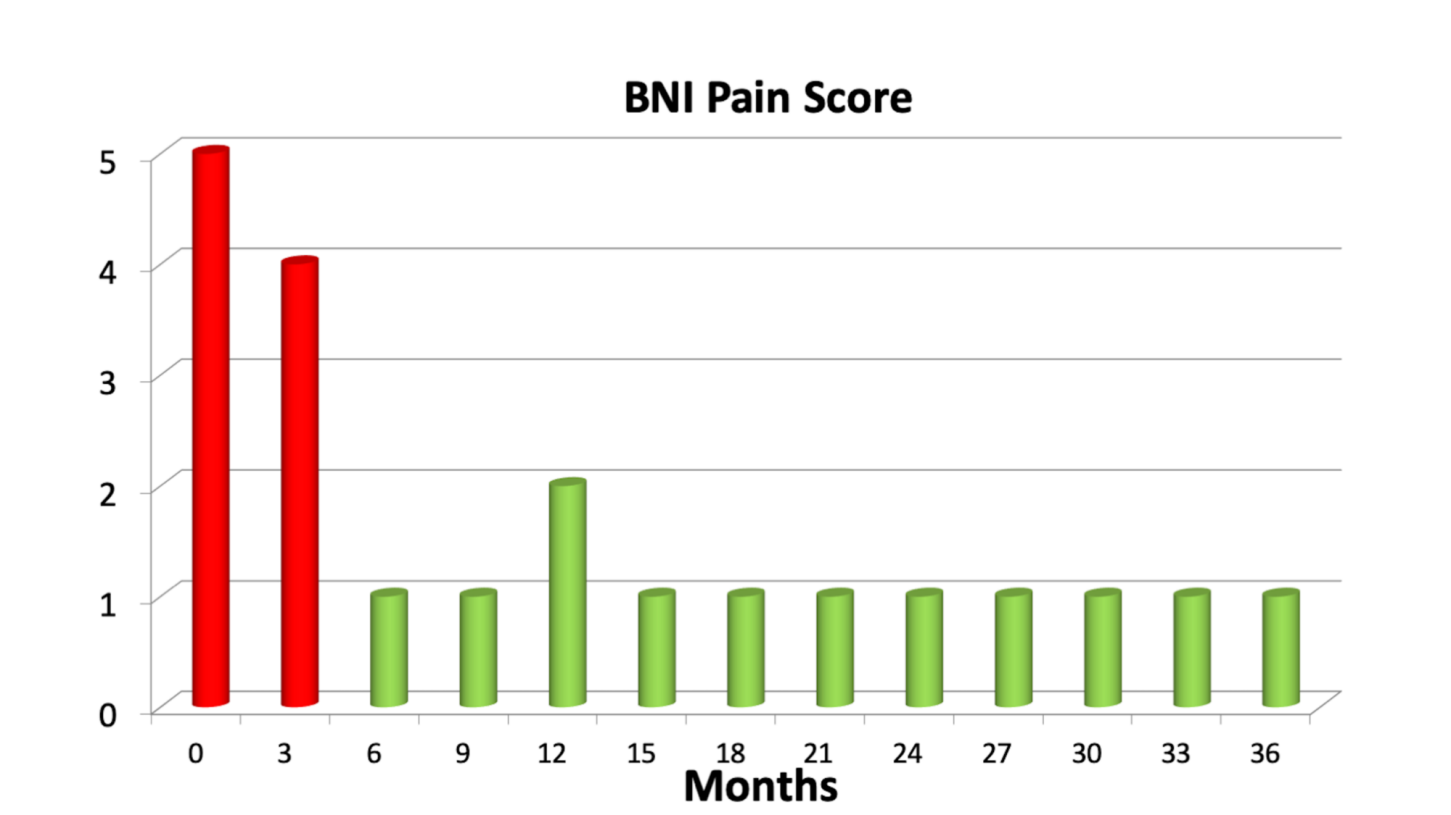
Mean Barrow Neurological Institute (BNI) pain categories in the first three years after treatment in a cohort of 343 patients

**Figure 3:**
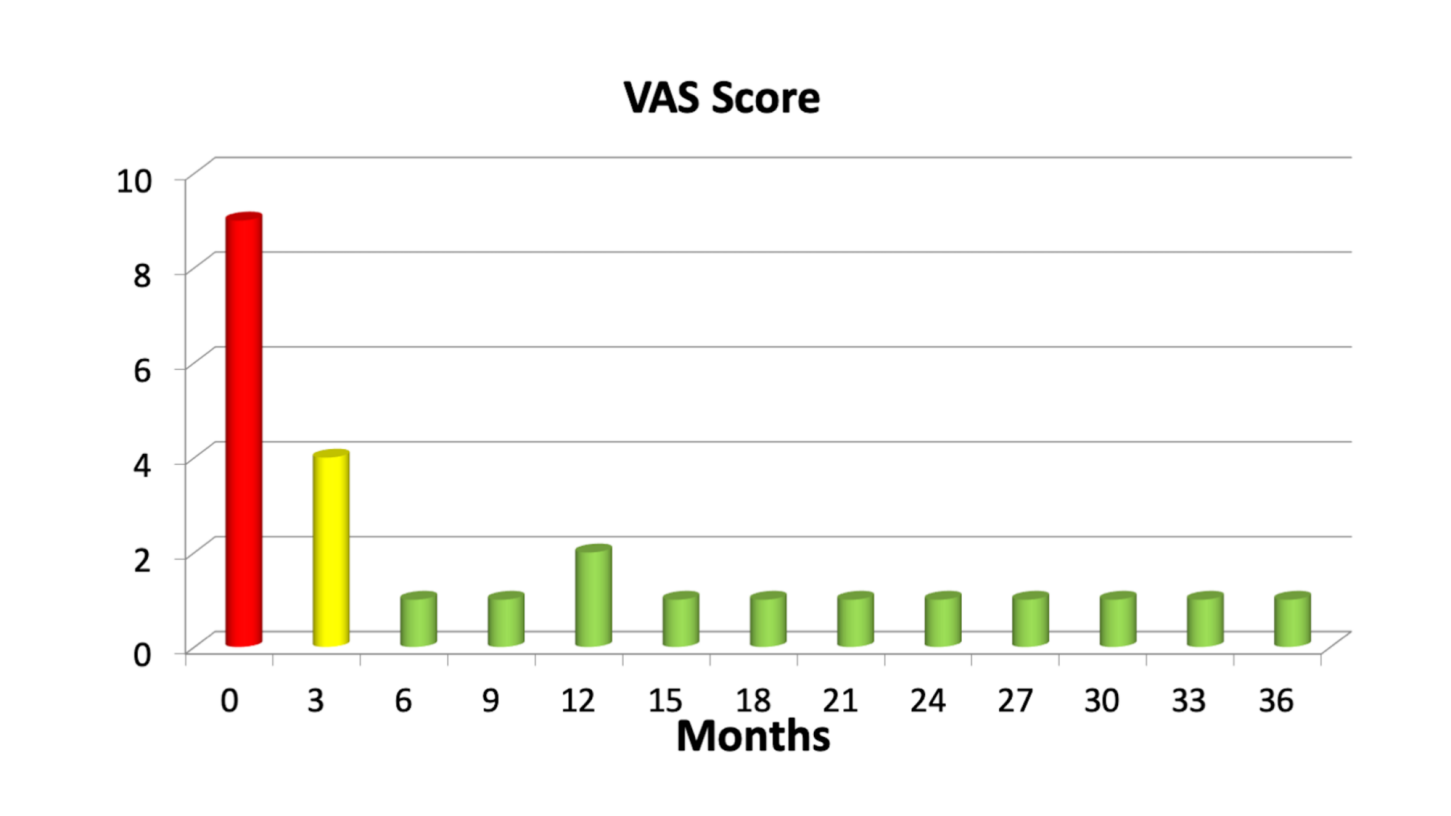
Mean visual-analog scale (VAS) pain score in the first three years after treatment in a cohort of 343 patients

Twenty-eight patients of 343 (8%) did not report any benefit at all after the procedure. Fifty-five (16%) pain-free patients experienced recurrent pain within three years from the treatment. The combined number of patients having no benefit (from the start or due to recurrent pain) three years after the treatment was 83 (24%). Figures 2, 3 show the rate of pain control after treatment.

Salvage therapy

**Figure 4:**
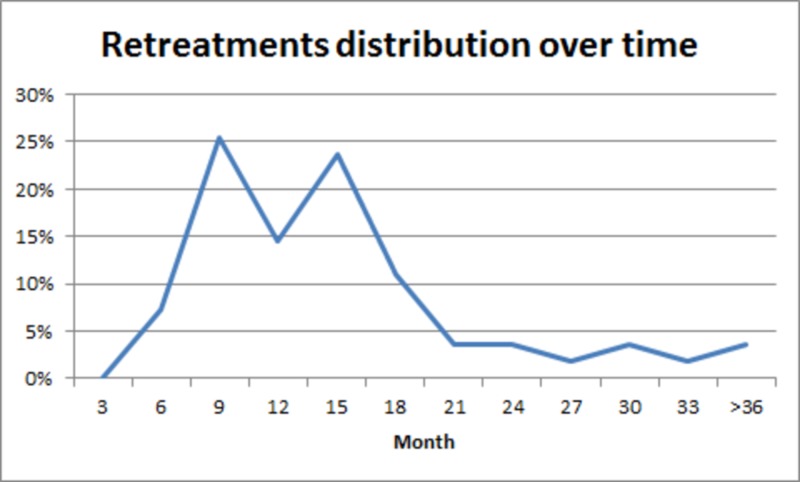
Temporal distribution of retreatments A bimodal distribution of retreatments is visible from the graphic. Peaks of retreatments are at 9 and 15 months after the first treatment. Then there is a drastic reduction in the number of retreatments.

Forty-four patients out of 343 (12.8%) required a second treatment during the observed period. Twenty patients (5.8%) who failed to achieve pain control after a minimum of six months and 24 of 316 pain-free patients (7.6%) who experienced recurrent pain within three years from the treatment underwent retreatment. The target remained approximately the same. Retreatments received, respectively, a median dose of 45 Gy (median prescription isodose of 83%) and a mean dose of 48.9 Gy (mean prescription isodose of 83.3%) over a median volume of 22.5 mm³ and a mean volume of 25.6 mm³. All 44 patients developed good pain control (BNI pain scores I-III) at the last follow-up, and they seem to achieve long-term pain control. The temporal distribution of retreatments shows a bimodal peak (Figure 4).

Sensory dysfunction and other complications

**Figure 5:**
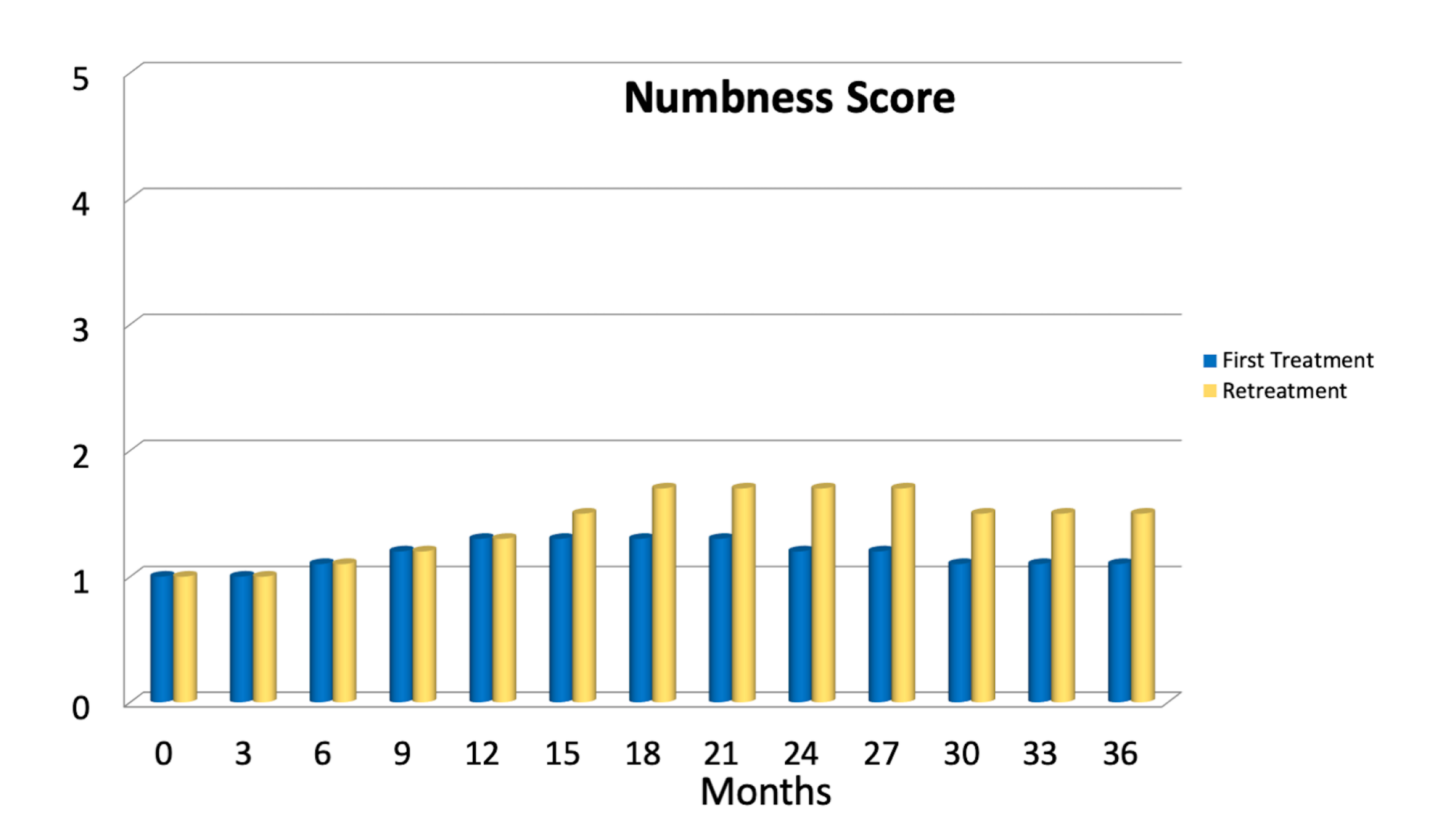
Mean Barrow Neurological Institute (BNI) Numbness score in the first three years after one single treatment or retreatment

Thirty-six months after treatment, 21 patients out of 343 (6.1%) reported the presence of facial numbness associated with bothering dysesthesias (Grade III/IV of the BNI numbness scale). Eighteen patients out of 21 (85.7%) developed a grade III sensory disturbance (somewhat bothering) after retreatment. Two patients reported BNI grade III numbness after the first treatment (0.6%). One further patient (0.3%) developed BNI numbness scale grade IV hypoesthesia after the first treatment. Mild, non-bothersome sensory disturbances, i.e., BNI numbness scale grade II, were reported by 48 patients (14%), with an overall rate of 20.1% (69 of 343 patients) developing some sensory disturbance, 6.1% of which were bothersome. On average, sensory complications developed after 16.4 ±8.7 months. Figure 5 shows the temporal distribution of BNI numbness scores. No further complications, such as temporal lobe radionecrosis, anesthesia dolorosa, lockjaw, weakness of the mandible, diplopia, dry-eye syndrome, keratitis, or hearing loss, were reported in this present series. However, four patients reported salivary drooling (in all cases associated with BNI grade II-III numbness).

## Discussion

The development of frameless radiosurgery for TN

The use of frameless non-isocentric stereotactic radiosurgery for the treatment of TN was introduced at Stanford by John Adler, the inventor of CyberKnife, and first reported by his fellow, Pantaleo Romanelli, in a study that was the first clinical demonstration of the accuracy and safety of frameless image-guided TN radiosurgery [14]. Almost immediate pain relief (within days) was found in this first cohort of patients following the delivery of a prescribed dose ranging from 65 to 70 Gy to a nerve segment up to 11 mm. The irradiation of such a long nerve segment, however, caused a high rate of bothersome numbness and prompted a reduction of dose and length of the nerve treated, leading to the treatment protocol reported here [15,16,19]. During early Cyberknife treatments for TN, a CT-cisternography was performed to provide safe targeting and avoid potentially unsafe MR-CT fusion in the earlier versions of the system. CT cisternography has been abandoned after the preliminary Stanford experience because later versions of MultiPlan-TPS provided a rather accurate CT-MR fusion capability. Also, bony landmarks, indicating the entrance of the trigeminal nerve root into the Meckel’s cave, are easily recognizable directly on a bone CT scan. The identification of these points greatly supports a precise co-registration of the CT with the MR sequence. Further experience was, therefore, based on MR-CT fusion targeting [17-24].

In 2008, Villavicencio et al. published data from a multicenter study illustrating the results of 95 patients who underwent CyberKnife radiosurgery [17]. This heterogeneous study included patients treated with widely different modalities (isocentric and non-isocentric) as well as variable doses and treatment volumes. The median dose used was 75 Gy. Certain variables were predictive of stable pain relief over pain recurrence, including the median maximum dose (77.5 versus 65 Gy), median minimum dose (64 versus 52 Gy), and median nerve length treated (4 mm versus 6 mm). After two years, 50% of the population had excellent results, but 47% suffered new facial numbness. An update from the Stanford series reported on 46 patients receiving a treatment delivered over a 6-mm segment of the nerve, with a mean marginal prescription dose of 58.3 Gy and a mean maximal dose of 73.5 Gy [16]. Symptoms disappeared completely in 39 patients (85%). After a mean follow-up period of 14.7 months, patient-reported outcomes were excellent in 33 (72%), good in 11 (24%), and poor in two patients (4%). Ipsilateral bothersome facial numbness (Grade III on the BNI numbness scale) was found in seven patients (15%). Further studies focusing on the general treatment of TN1, TN2, and multiple sclerosis (MS) related TN provided similar outcomes [18,20-23].

Our group has recently published a prospective study reporting the long-term outcomes of a cohort of 138 TN patients treated with frameless image-guided radiosurgery [13]. Median follow-up was 52.4 months; median target length 5.7-mm; median target volume 40 mm³; median prescription dose 60 Gy ( to the 80% isodose line). The actuarial pain control rate scored using the BNI classification at 6, 12, 24, and 36 months was, respectively, 93.5%, 85.8%, 79.7%, and 76%. A second treatment, due to primary treatment failure or recurrent pain, was offered to 33 patients (24%). Sensory complications have also been assessed using BNI scores. Overall, the rate of sensory disturbances was 18.1%. BNI grade II (not bothersome) hypoesthesia was reported by 18 patients (13%), with 10 patients receiving two treatments and one patient having three treatments. Six patients (4.3%) developed BNI grade III (somewhat bothersome) hypoesthesia, all after retreatment. One patient (0.7%) developed BNI grade IV dysfunction. The average delay for the appearance of sensory complications was 16.4 months after irradiation. Nerve length (<6 mm vs. 6 mm), smaller nerve volume (<30 mm3 vs.>30 mm3), and lower prescription dose (<58 vs.>58 Gy) were found to be associated with treatment failure. Re-irradiation independently predicted sensory disturbance (P < .001). The pain control rates became stable after three years and remained so for the following three years. 

Clinical outcomes of stereotactic radiosurgery for TN

This series represents the largest cohort to date reporting about frameless radiosurgery for TN. The treatment was delivered using the CyberKnife, a robotic image-guided linear accelerator providing non-isocentric beam delivery. Homogeneous dose distribution over an extended segment of the trigeminal nerve was made possible by the use of this radiosurgical device. A treatment protocol delivering 60 Gy to a 6 mm retrogasserian segment of the nerve was delivered to all the patients. The maximum dose could not exceed 75 Gy. A detailed assessment of clinical outcomes was performed over the three years following the treatment of 343 patients. According to our previous prospective study reporting on 138 patients, rates of pain control became stable three years after the treatment and remained so over the following three years [13]. We, therefore, retrospectively analyzed the clinical outcomes during the first three years after the treatment in the much larger cohort reported here. The pain relief rate six months after the treatment was 92%, with 76% of patients experiencing stable benefit (BNI class I-III) three years after the treatment (Figure 1).

These results are consistent with those reported by similar studies reporting about frame-based radiosurgery [1-12]. The two studies reporting about the largest patient populations show a high rate of pain control [1,4]. The Marseille group illustrated the results of 497 patients with primary TN after one year of follow-up. Of these patients, 91% achieved pain freedom in a short time. Pain recurred in 34.4% of patients [1]. In their report on 448 patients, Marshall et al. described satisfactory pain control in 86% and recurrence in 28% after three months of follow-up [4]. 

Sensory complications have been found in 20.1% of our patients, which is also in line with the literature. Regis et al. have recently updated their original study of 497 patients, reporting the long-term results of this large group of patients. The rate of new sensory disturbances was 20.4% [6]. In our series, only 21 patients (6.1%) developed bothersome hypoesthesias, with a very substantial number (18 out of 21) having been treated two times. Therefore, the risk of bothersome sensory deficit after a single treatment can be calculated as less than 1% following frameless Cyberknife radiosurgery using a retrogasserian target and specific dose/volume constraints.

The dose constraints we prescribe to the brainstem and gasserian ganglion cannot exceed 15 and 25 Gy, respectively. Frame-based irradiation using the gamma-knife can be associated with higher brainstem doses and higher rates of sensory complications, especially when maximum doses exceed 80 Gy [1-12].

Novel findings

The current report substantially confirmes the earlier findings on a larger number of patients (343) with a follow-up of three years. Frameless image-guided targeting of a 6-mm segment of the trigeminal nerve with a prescribed dose of 60 Gy and a maximum dose not superior to 75 Gy is a safe and effective technique, achieving stable pain control with minimal risks. Sensory complications are rare and typically affect patients receiving multiple treatments. It could be argued that a second treatment may not be warranted due to the higher risk of sensory complications. However, the overall risk of developing hypoesthesia and paresthesias remains relatively small even after repeated treatment. The absence of any other neurological complication after frameless radiosurgery, if compared with the potential complications of medical therapy (including liver, renal, and bone marrow failure) and other non-medical approaches such as percutaneous procedures or microvascular decompression, prompts careful scrutiny while evaluating management options for patients with recurrent pain. It also needs to be mentioned that over time, the doses delivered during the second treatment have been reduced from 60 to 45 Gy, thus achieving a substantial reduction of rate and intensity of sensory complications. A further paper will investigate in detail the relation between integral dose to the nerve and sensory outcomes.

The current study confirms that CyberKnife frameless radiosurgery delivering homogeneous dose to an elongated segment of the trigeminal nerve is associated with a high rate of pain control and minimal risk to develop hypoesthesia and paresthesias after a single treatment. These favorable outcomes could be related to the use of a non-isocentric technique. As opposed to isocentric treatment, non-isocentric dose delivery provides the ability to define the target volume according to the individual anatomy and to deliver a homogeneous dose over the target. Isocentric planning requires the use of 4 mm collimators, thus restricting the irradiated segment to 4 mm. A second collimator can be placed, which is likely to create a hot spot where the two collimators dose distributions overlap. Increased morbidity and no improvement in pain control were found after isocentric treatments placing two collimators over the trigeminal nerve [24]. Non-isocentric dose delivery allows to tailor the irradiation based on the trigeminal nerve anatomy, thus providing a homogeneous treatment over a nerve segment longer than 4 mm.

Focus on treatment planning

**Figure 6:**
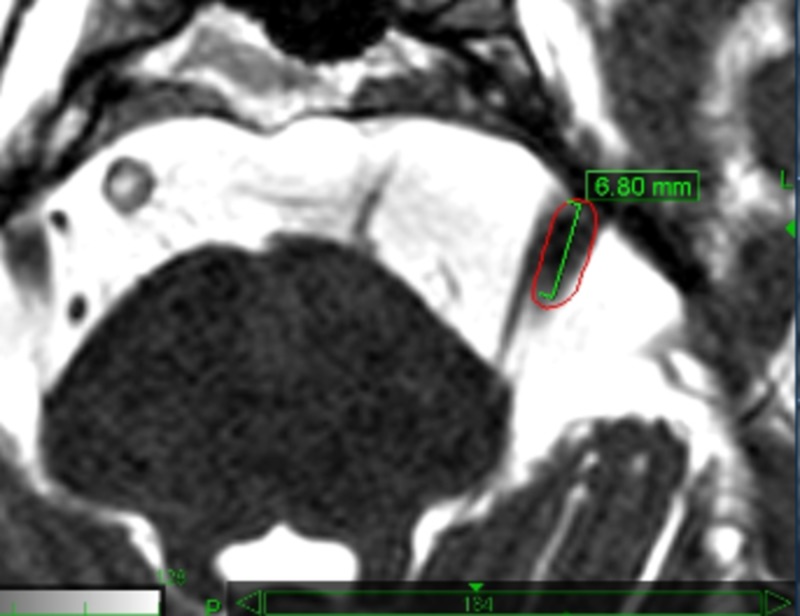
The motor root of the left trigeminal nerve is medial to the sensory root. The target volume (red line) encloses the sensory root alone. The segment of the sensory root is 6.8 mm long.

**Figure 7:**
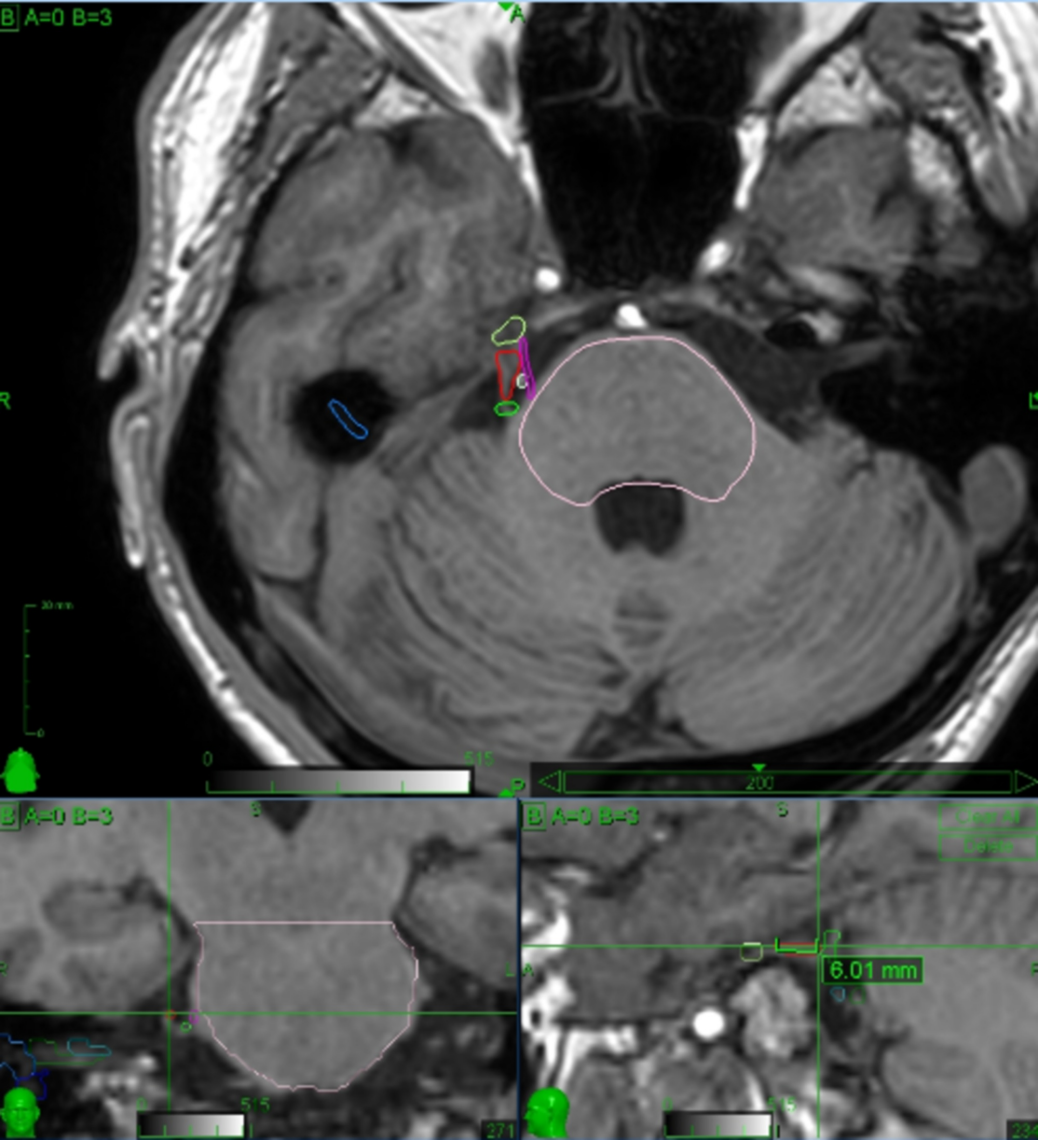
3D reconstruction of the target (sensory root of the affected trigeminal nerve) and critical structures nearby (brainstem, gasserian ganglion, trigeminal motor root.offending vessels) The sensory root is drawn using a red line, the motor root with a purple line, the brainstem with a pink line, the gasserian ganglion with a yellow line. There are two offending vessels, one located at the root entry zone (green line) and one dissecting the sensory and motor roots (white line). Axial view is above, coronal and sagittal views are below, respectively on the left and right side. Crosshairs are placed at the proximal end of the target. As shown on the sagittal view, the target includes a 6.01 mm segment of the sensory root.

**Figure 8:**
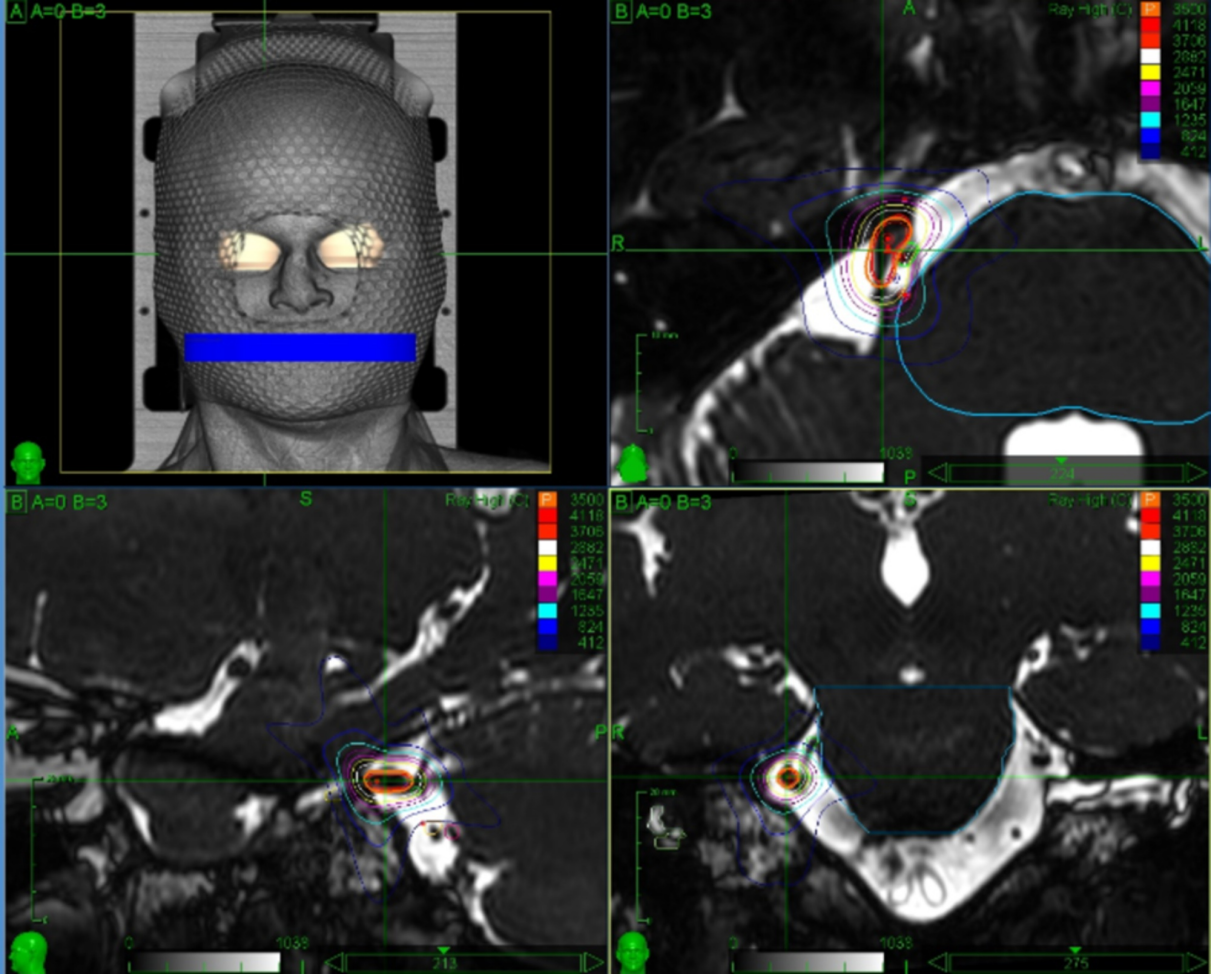
Treatment planning developed over the anatomy shown in Figure 7 This plan shows how the careful identification of the anatomic structures involved can grant a certain degree of dose sparing on the trigeminal motor roots and the offending vessels as well as on the gasserian ganglion and brainstem while preserving the intended prescribed dose to the sensory root.

Careful planning by an expert physician is crucial to enhance treatment safety and to provide the best clinical outcomes. Detailed knowledge of the involved anatomy is extremely important in order to avoid complications. Critical structures drawn by the treating physicians include the brainstem, gasserian ganglion, cranial nerves VII and VIII, cochlea and labyrinth. The offending vessel, if present, is also drawn, thus avoiding the inclusion within the target volume. Special care is taken in the identification of the affected trigeminal nerve, which is often compressed and distorted by the offending vessel, or atrophic due to previous invasive treatments. The motor root of the trigeminal nerve lies medial and slightly superior to the sensory root and can be identified and drawn as a separate structure, thus receiving lower doses than the sensory root (Figures 6, 7). Figure 8 shows the treatment planning developed over the anatomy shown in Figure 7.

**Figure 9:**
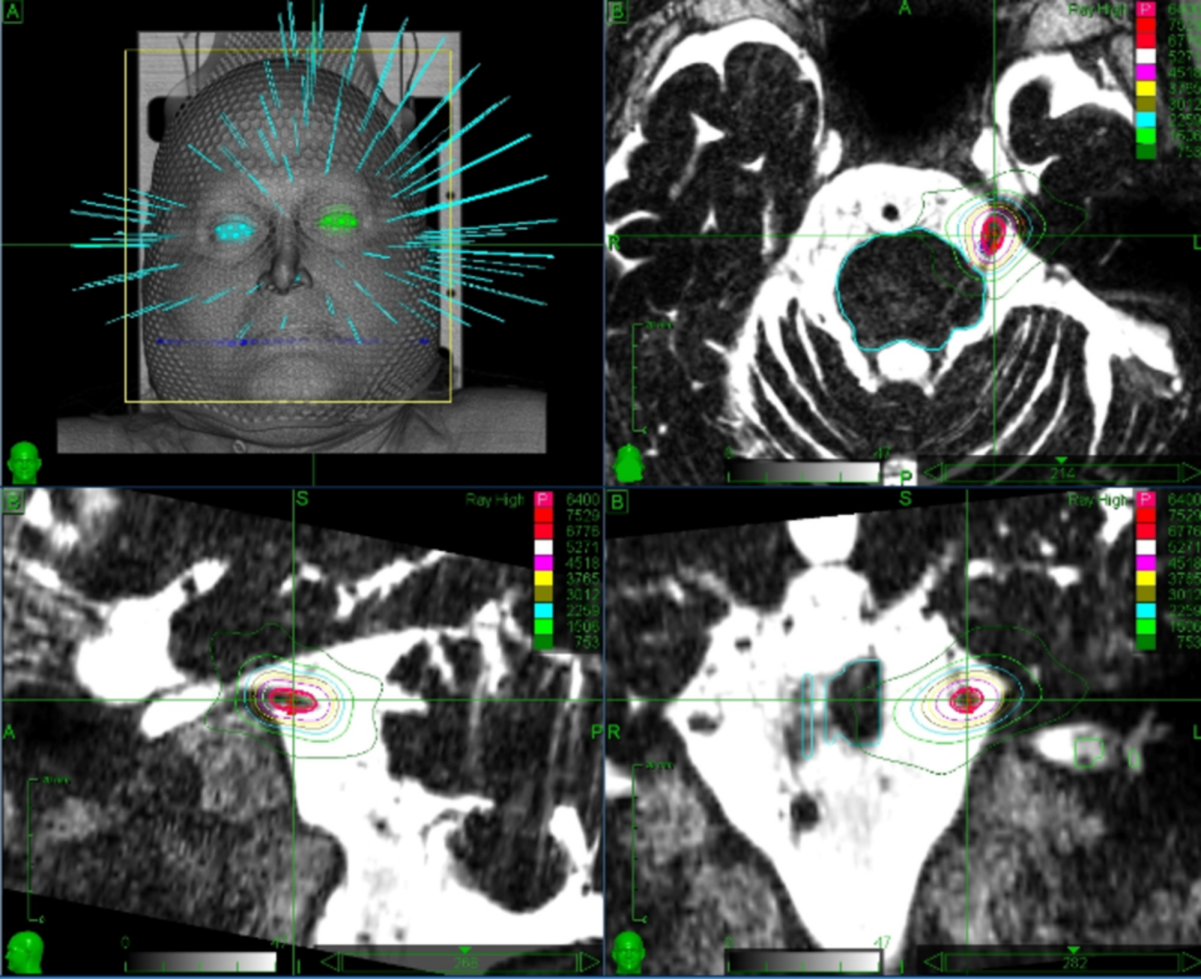
Treatment planning for a trigeminal neuralgia case The target includes a 6 mm long retrogasserian segment of the left trigeminal nerve. The dose delivered is 60 Gy, prescribed to the 80% isodose. Treatment volume is 0.03 cc.

Figure 9 shows a typical treatment plan delivering 60 Gy (prescribed to the 80% isodose) to the retrogasserian segment of the affected trigeminal nerve. Great care is taken to orient the major axis of the isodoses in a tangential fashion to the major axis of the nerve, thus maintaining most of the irradiation over the cisternal spaces with consequent sparing of the brainstem and gasserian ganglion. 

Limits of the study and further work ahead

This study is limited by the retrospective design and the lack of prolonged follow-up. A 10 years follow-up is desirable for an assessment of the validity of the frameless technique reported here. Also, we did not make a dedicated analysis to find out if MS or atypical pain (TN2) influenced the clinical outcomes. Further work will be devoted to provide a 10 years follow-up on the available patients and to assess the relationship between integral dose received by the treated nerve and clinical outcomes, with a special reference to cumulative doses after retreatments. Clinical outcomes of frameless Cyberknife treatments delivered to MS and TN2 patients will be discussed in further reports.

## Conclusions

Stereotactic radiosurgery is emerging as a valid first-line treatment option for TN. This study on a large number of patients undergoing frameless radiosurgery using the Cyberknife shows that this technique is safe and effective. Using our constraints for dose, volume of the nerve, and dose to the brainstem and gasserian ganglion, the incidence of bothersome sensory complications was low, whereas a durable pain control was achieved in 76% of patients. The rarity of bothersome complications and the fact that frameless radiosurgery represents the less invasive technique for the surgical treatment of TN provide a particularly favorable profile to this technique as compared with other medical and non-medical treatments.
